# Trajectory of cognitive function and quality of life following stenotic aortic valve procedures

**DOI:** 10.3389/fcvm.2026.1659733

**Published:** 2026-02-06

**Authors:** Marco Ranucci, Giuseppe Maria Raffa, Luca Ranucci, Luca Brischigiaro, Stefano Casalino, Vittoria Mazzotta, Martina Anguissola, Oronzo Catalano, Matteo Montorfano, Luca Angelo Ferri, Sabrina Ferrante, Maria Teresa La Rovere, Giulio Stefanini, Ottavia Cozzi, Lorenzo Menicanti

**Affiliations:** 1 Department of Cardiovascular Anesthesia and Intensive Care, IRCCS Policlinico San Donato, Milan, Italy; 2 Department for the Treatment and Study of Cardiothoracic Diseases and Cardiothoracic Transplantation, IRCCS-ISMETT, Palermo, Italy; 3 Cardiac Surgery Unit, Department of Precision Medicine in Medical Surgical and Critical Area (Me.Pre.C.C.), University of Palermo, Palermo, Italy; 4 Department of Cardiac Rehabilitation, IRCCS Istituti Clinici Scientifici Maugeri, Pavia, Italy; 5 School of Medicine, Vita-Salute San Raffaele University, Milan, Italy; 6 Interventional Cardiology Unit, IRCCS San Raffaele Scientific Institute, Milan, Italy; 7 Department of Cardiac Rehabilitation, IRCCS Istituti Clinici Scientifici Maugeri, Montescano, Italy; 8 Cardio Center, IRCCS Humanitas Research Hospital, Rozzano, Milan, Italy; 9 Cardiac Network of IRCCS and Department of Cardiac Surgery, IRCCS Policlinico, Milan, Italy

**Keywords:** aortic valve stenosis, neurocognitive function, quality of life, surgical aortic valve replacement, transcatheter aortic valve implantation

## Abstract

**Aims:**

Severe aortic valve stenosis can be treated with either surgical valve replacement (SAVR) or transcatheter valve implantation (TAVI). The choice between these two strategies is guided by the age and clinical profile of the patient. Our study aims to verify the hypothesis that including health-related quality of life (HRQL) as an outcome measure may be relevant to therapeutic choice.

**Methods:**

This prospective observational study included 806 patients aged 65–80 years who received either SAVR or TAVI. HRQL was assessed using the SF-12 questionnaire before and after the procedure (1–4 years of follow-up). Propensity score matching was applied to account for baseline differences between groups, resulting in two matched groups of 92 patients each. A subgroup of 80 patients received a neurocognitive function assessment before the procedure and at 2–3 months of follow-up.

**Results:**

At 1-year follow-up, no significant differences were observed between SAVR and TAVI in terms of the mental and physical components of the SF-12, with both procedures resulting in significant improvements in HRQL in both the prematching and propensity-matched populations. In the prematching population, at 4-years follow-up and after adjustment for potential confounders, the cumulative risk of HRQL deterioration did not differ significantly between SAVR and TAVI for the mental component (hazards ratio 1.03, 95% confidence interval 0.69–1.56, *P* = 0.881), while it was significantly higher (hazards ratio 1.89, 95% confidence interval 1.02–3.59, *P* = 0.045) for the physical component in the TAVI group. After propensity score matching, these results were confirmed, with no significant differences in the mental component (hazards ratio 1.14, 95% confidence interval 0.65–2.00, *P* = 0.640) and a significantly higher risk of worsening in the physical component in the TAVI group (hazards ratio 3.91, 95% confidence interval 1.46–10.5, *P* = 0.007). Early cognitive impairment was associated with a significantly higher risk of deterioration in the mental component at 1-year follow-up (relative risk 3.2, 95% confidence interval 1.18–8.94, *P* = 0.023).

**Conclusion:**

In a real-world scenario, no differences in quality of life were observed between SAVR and TAVI at 1-year follow-up; conversely, at 4-year follow-up, the physical component of HRQL appeared to be better preserved in patients undergoing SAVR.

## Introduction

Severe aortic valve stenosis (AS) is the most common valvular disease in the elderly (1). Current therapeutic options are primarily based on surgical aortic valve replacement (SAVR) and transcatheter aortic valve implantation (TAVI). SAVR allows implementation of either a mechanical or biological valve prosthesis, while TAVI is feasible only with a biological prosthesis. The decision on which strategy to apply depends on several factors, with patient age and the presence of comorbidities being the primary considerations. Initially, TAVI was preferred in elderly (>80 years) patients at high surgical risk; presently, patients at low (2, 3) or intermediate (4) surgical risk are considered suitable for TAVI.

The majority of studies comparing SAVR and TAVI have focused on outcome measures related to mortality and major complications (such as stroke, heart failure, and re-hospitalization). From an indications viewpoint, there is presently a discrepancy between the European (5) and US (6) guidelines for the management of AS: European guidelines recommend SAVR for patients <70 years in the absence of major comorbidities; conversely, US guidelines suggest SAVR for patients <65 years. Given this discrepancy and considering the differences between SAVR and TAVI in terms of costs, resource utilization, and invasiveness, it is reasonable to include assessment of health-related quality of life (HRQL) in the decision-making process when deciding whether to refer a patient to SAVR or TAVI. This consideration is especially important in patients with limited life expectancy, for whom achieving increased HRQL within a reasonable postprocedural timeframe is mandatory. Data on HRQL are available but limited to studies with relatively short follow-up durations (3, 7–9), selected patient populations (3, 7, 10), and small sample sizes (7, 9).

In 2018, under the auspices of the Italian Ministry of Health and within the Cardiac Network of the Italian Clinical Research Hospitals (IRCCS), data on SAVR and TAVI procedures began to be collected in a national registry (OUTcomes evaluation of current therapeutic STrategies for severe Aortic valve steNosis and the ageING population in ITALY: OUTSTANDING-ITALY). The purpose of the OUTSTANDING-ITALY registry is to prospectively collect data on SAVR and TAVI procedures to offer a real-world assessment of procedural outcomes. Within this registry, several institutions collected data on HRQL and cognitive function to test the hypothesis that the patient-reported outcome (PRO) of HRQL does not differ between SAVR and TAVI at long-term follow-up. A secondary endpoint was to verify the hypothesis that early cognitive impairment after SAVR or TAVI procedures may constitute a risk factor for subsequent worsening in the mental component of HRQL.

## Methods

All patients were enrolled prospectively according to the study design of the OUTSTANDING-ITALY registry, which is funded by the Italian Ministry of Health. The study was approved by the local ethics committee of San Raffaele Hospital (OSR 14/12/2017, protocol number 298/2017) and subsequently amended to include the assessment of neurocognitive function. All patients provided written informed consent for participation.

### Patient population and data collection

Patients were enrolled and had at least one follow-up assessment at five institutions within the Cardiac Network of IRCCS: IRCCS Policlinico San Donato (San Donato Milanese, 371 patients), IRCCS San Raffaele (Milano, 10 patients), IRCCS Humanitas (Rozzano, 32 patients), IRCCS Fondazione Maugeri (Pavia and Montescano, 18 patients), and IRCCS ISMETT (Palermo, 69 patients). They underwent either SAVR or TAVI for the treatment of severe aortic valve stenosis. The criteria for determining which procedure to use for the patient were based on the standard policy of the Institution, generally following the guidelines in effect at the time of the procedure. The minimum age for study enrollment was set at 66 years. Data collection included preprocedural, procedural, and postprocedural factors included in the OUTSTANDING-ITALY registry. HRQL was assessed using the Medical Outcomes Study Short-Form 12 (SF-12) questionnaire (11), which was administered to the patients by a dedicated psychologist through a direct interview on the day before the procedure (baseline assessment) and during follow-up via telephone interview. Follow-up assessments were scheduled at 1, 2, 3, and 4 years after the procedure. However, due to the COVID-19 pandemic, many patients were lost to follow-up at 3 years; therefore, data from this time point were not considered. The SF-12 produced a mental component summary (MCS) and a physical component summary (PCS). In a subgroup of 80 patients (all treated at IRCCS San Donato), a neurocognitive assessment was performed immediately before the procedure and again at 2–3 months of follow-up. Professional psychologists (LR and LB) performed the cognitive assessment, which was based on the Montreal Cognitive Assessment (MOCA). The MOCA is a screening test designed to evaluate cognitive function and to detect mild cognitive impairment (12, 13). It is a 30-point test that assesses multiple cognitive domains: memory (immediate and delayed recall), visuospatial abilities, executive functions, attention, concentration, language, and spatial and temporal orientation. The complete results of neurocognitive changes after the procedure have already been published elsewhere (14).

### Statistics

Data are expressed as mean (standard deviation), median (interquartile range), or number (percentage), as appropriate. Comparisons between the SAVR and TAVI groups were performed using Pearson's chi-square test for differences between binary variables and Student's *t*-test or non-parametric tests for differences between continuous variables. A repeated-measures analysis of variance (ANOVA) was used to test differences in SF-12 scores at the 1-year follow-up, assessing within- and between-group differences.

Our study focuses on the assessment of changes in HRQL and cognitive function, as measured by the MOCA test. Therefore, a reliable definition of clinically relevant changes is of paramount importance. For this purpose, we applied the reliable change index (RCI) to stratify the changes into three categories: improved, unchanged, or worsened. Briefly, the RCI was calculated as follows: [(X2−X1)−(M2−M1)]/SED, where X1 = observed first test score, X2 = observed second test score, M1 = group mean first test score, M2 = group mean second test score, and SED = standard error of the difference corrected for the test–retest correlation coefficient *r* (15, 16). The RCI provides a confidence interval for the mean differences in scores. For all RCI calculations, statistical significance was set at an alpha value of 0.05 (two-tailed). Therefore, a “reliable improvement” was defined as an RCI exceeding +1.96, whereas a “reliable worsening” was defined as an RCI below −1.96.

To investigate the endpoints of our study, the following analyses were performed:

a. The patient population was tested for between-group differences. Given the anticipated differences in patient characteristics, two groups (SAVR and TAVI) were identified using 1:1 propensity score matching. The propensity score matching model included all covariates associated with the type of procedure at *P* <0.1. Differences between groups before and after matching were assessed using the standardized mean difference (SMD). The matching procedure was considered adequate for an SMD <0.10. The cumulative risk of worsening HRQL was assessed using Kaplan–Meier analysis and the log-rank test. HRQL analyses were applied to both pre- and postmatching populations.
b. Changes in SF-12 MCS/PCS scores from baseline to 1-year follow-up were expressed as absolute values, assessing within- and between-group differences, and as RCI-based categories (improved, unchanged, and worsened), assessing the differences using Pearson's chi-square test.
c. The cumulative risk of experiencing a clinically relevant worsening of the SF-12 MCS/PCS throughout the follow-up period was addressed using Kaplan–Meier analysis, with between-group comparisons performed using the log-rank test. Multivariable adjustment for potential confounders (including preprocedural factors) was conducted using Cox regression analysis. For the purpose of this analysis, an event (worsening of the SF-12 MCS/PCS) was recorded at any specific time of follow-up, without however censoring patients who developed the event. This approach allowed potential improvements to be captured in patients who experienced an event but subsequently recovered to an unchanged or even improved condition. This analysis, focusing on relative changes in HRQL rather than absolute values, was anticipated to reduce the potential bias arising from preprocedural differences between the SAVR and TAVI groups in the overall patient population.
d. The correlation between changes in neurocognitive function and HRQL was examined in a subgroup of 80 patients and was limited to the 1-year follow-up. Early neurocognitive changes (worsened or unchanged/improved based on MOCA results) were compared to the 1-year SF-12 MCS using Pearson's chi-square test and logistic regression analysis to account for the type of procedure (SAVR or TAVI). For these analyses, relative risk or odds ratios with 95% confidence intervals were reported.

The primary outcome of the study was the change in HRQL, assessed using the SF-12 PCS and MCS, after the aortic valve procedure, with a maximum follow-up of 48 months. The secondary outcome was the assessment of the correlation between early postprocedural changes in cognitive function, measured by the MOCA test, and subsequent changes in the SF-12 MCS.

All statistical analyses were performed using computerized packages (SPSS 20.0, IBM, Chicago, IL, USA; GraphPad, GraphPad Software, Inc., San Diego, CA, USA; and MedCalc, MedCalc Software, Ostend, Belgium). A two-tailed *P* value < 0.05 was considered significant for all statistical tests.

## Results

Overall, 806 patients underwent a preprocedural assessment of HRQL between September 18, 2018 and November 24, 2024. Of this patient population, 306 patients failed to complete any of the HRQL follow-up assessments after discharge from the hospital. Patients lost to follow-up included 220 in the TAVI group (42%) and 86 in the SAVR group (31%). The final study population therefore comprised 500 patients (179 SAVR and 321 TAVI), whose characteristics are reported in Table 1. As expected, patients in the TAVI group exhibited a significantly more severe profile than those in the SAVR, including older age, lower left ventricular ejection fraction, and a higher prevalence of risk conditions such as prior cerebrovascular accident and prior vascular or percutaneous interventions. HRQL was significantly (*P* = 0.001) worse in the physical domain among TAVI patients, whereas no differences were observed in the mental domain.

**Table 1:** Preprocedural pattern of the patient population.

Variable	Overall	SAVR	TAVI	*P*
	(*N* = 500)	(*N* = 179)	(*N* = 321)	
Demographics				
Age (years)	78.5 (6.5)	73.3 (4.8)	81.3 (5.5)	0.001
Gender male	253 (50.6)	90 (50.3)	163 (50.9)	0.926
Body mass index (kg/m^2^)	27.0 (4.5)	27.6 (4.3)	26.6 (4.5)	0.025
Baseline conditions				
Hemoglobin (g/dL)	13.0 (4.6)	13.1 (1.7)	13.0 (5.6)	0.944
Left ventricular ejection fraction	58.4 (10.6)	62.0 (10.1)	58.1 (10.6)	0.001
Serum creatinine (mg/dL)	1.14 (0.98)	1.06 (1.2)	1.19 (0.82)	0.157
Comorbidities				
Arterial hypertension	397 (79.4)	145 (81.1)	252 (78.5)	0.245
Diabetes	138 (27.6)	44 (24.4)	94 (29.3)	0.294
Serum creatinine (mg/dL)	1.14 (0.98)	1.06 (1.2)	1.19 (0.82)	0.157
Dialysis	11 (2.2)	2 (1.1)	9 (2.8)	0.342
Previous myocardial infarction	45 (9)	12 (6.9)	33 (10.3)	0.253
Unstable angina	10 (2)	7 (4)	3 (1)	0.041
Coronaropathy	78 (15.6)	25 (14.3)	53 (16.6)	0.522
Congestive heart failure	83 (16.6)	31 (17.8)	52 (16.5)	0.707
Hypercholesterolemia	66 (13.2)	17 (9.7)	49 (15.4)	0.129
Hypertriglyceridemia	24 (4.8)	11 (5.9)	13 (4.1)	0.471
Syncope	36 (7.2)	13 (7.4)	23 (7.2)	0.935
COPD	56 (11.2)	23 (13.1)	33 (10.3)	0.376
Oxygen dependency	14 (2.8)	4 (2.3)	10 (3.1)	0.779
Cerebrovascular accident	39 (7.8)	7 (4)	32 (10)	0.022
Peripheral arteriopathy	34 (6.8)	8 (4.5)	26 (8.3)	0.140
Previous vascular surgery	29 (5.8)	4 (2.3)	25 (8.1)	0.009
Previous cardiac surgery	54 (10.8)	14 (8)	40 (12.6)	0.133
Previous PCI	89 (17.8)	13 (7.4)	76 (23.8)	0.001
Medications				
ACE inhibitors	194 (38.8)	81 (45.6)	113 (35.3)	0.038
Beta-blockers	255 (55.1)	98 (55)	177 (55.1)	0.986
Calcium channel blockers	121 (24.2)	44 (24.4)	77 (24.1)	0.940
Statins	297 (59.4)	96 (53.1)	201 (62.7)	0.062
Sartanes	125 (25)	42 (23.2)	83 (25.9)	0.617
Warfarin	133 (26.7)	42 (23.3)	91 (28.3)	0.341
P2Y12 inhibitors	91 (18.3)	15 (7)	76 (23.5)	0.001
Insulin	32 (6.3)	16 (9.2)	16 (4.9)	0.116
Antiarrhythmics	33 (6.7)	7 (4)	26 (7.9)	0.193
Diuretics	240 (48)	75 (41.7)	165 (50.9)	0.107
Oral antidiabetics	56 (11.3)	18 (10.3)	38 (11.7)	0.735
Salicylates	252 (50.3)	85 (47.2)	167 (51.9)	0.412
Direct oral anticoagulants	39 (7.8)	7 (3.2)	32 (9.8)	0.025
Quality of life				
SF-12 mental component	49.4 (9.5)	49.1 (9.6)	49.2 (9.4)	0.614
SF-12 physical component	40.7 (9.7)	42.6 (10)	39.6 (9.4)	0.001

Data are presented as number (%) or mean (standard deviation); ACE, angiotensin-converting enzyme; COPD, chronic obstructive pulmonary disease; MET, metabolic equivalent of task; PCI, percutaneous intervention; SAVR, surgical aortic valve replacement; TAVI, transcatheter aortic valve implantation.

### Prematching analyses

#### One-year follow-up

A total of 447 patients were available for HRQL assessment at 1 year after the procedure, with a 10% loss to follow-up. The SF-12 MCS score was 49.8 ± 8.9 in the SAVR group (baseline 48.6 ± 9.6) and 50.2 ± 8.0 in the TAVI group (baseline 49.2 ± 9.4), with a significant within-group improvement (*P* = 0.036). No significant between-group difference was observed (*P* = 0.066). The SF-12 PCS score was 47.7 ± 9.9 in the SAVR group (baseline 41.1 ± 9.9) and 45.8 ± 9.3 in the TAVI group (baseline 39.7 ± 9.4), with significant within-group improvement (*P* = 0.001) and no significant between-group difference (*P* = 0.240).

**Figure 1:**
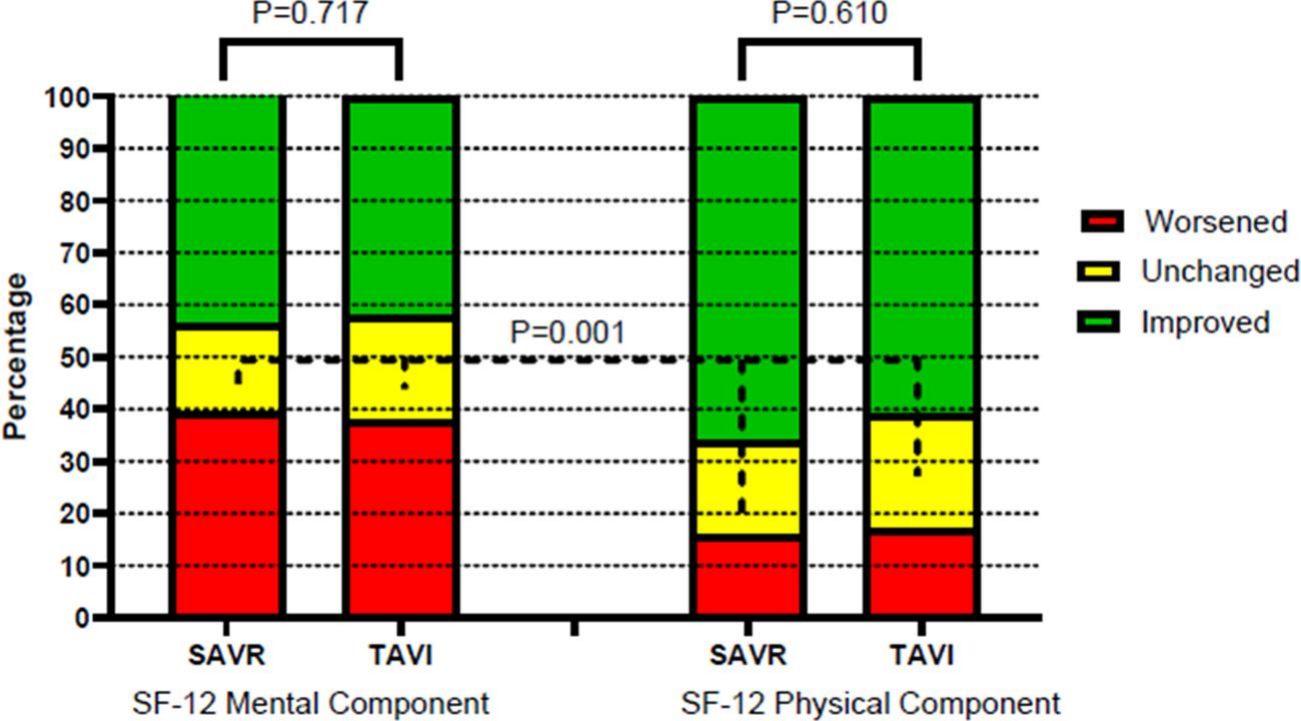
One-year changes in mental and physical components of health-related quality of life (prematching). SAVR, surgical aortic valve replacement; TAVI, transcatheter aortic valve implantation.

Changes in HRQL, defined based on the RCIs, were addressed at 1-year follow-up (Figure 1). Overall, the changes were not significantly different between the TAVI and SAVR groups. In both groups, a reliable worsening of the SF-12 MCS was observed in 38% and 39% of patients, respectively, while the SF-12 PCS showed a significantly lower rate of worsening (17% and 16%, respectively) compared with the SF-12 MCS (*P* = 0.001).

In the SAVR group, the only preprocedural factor associated with worsening of the SF-12 MCS was serum creatinine level (*P* = 0.05), while in the TAVI group, preprocedural factors associated with worsening of the SF-12 MCS were serum creatinine (*P* = 0.035), hypercholesterolemia (*P* = 0.025), and hypertriglyceridemia (*P* = 0.017). In the SAVR group, the only preprocedural factor associated with worsening of the SF-12 PCS was unstable angina (*P* = 0.005), whereas we could not find any preprocedural factor significantly associated with worsening of the SF-12 PCS in the TAVI group.

#### Long-term follow-up

**Figure 2:**
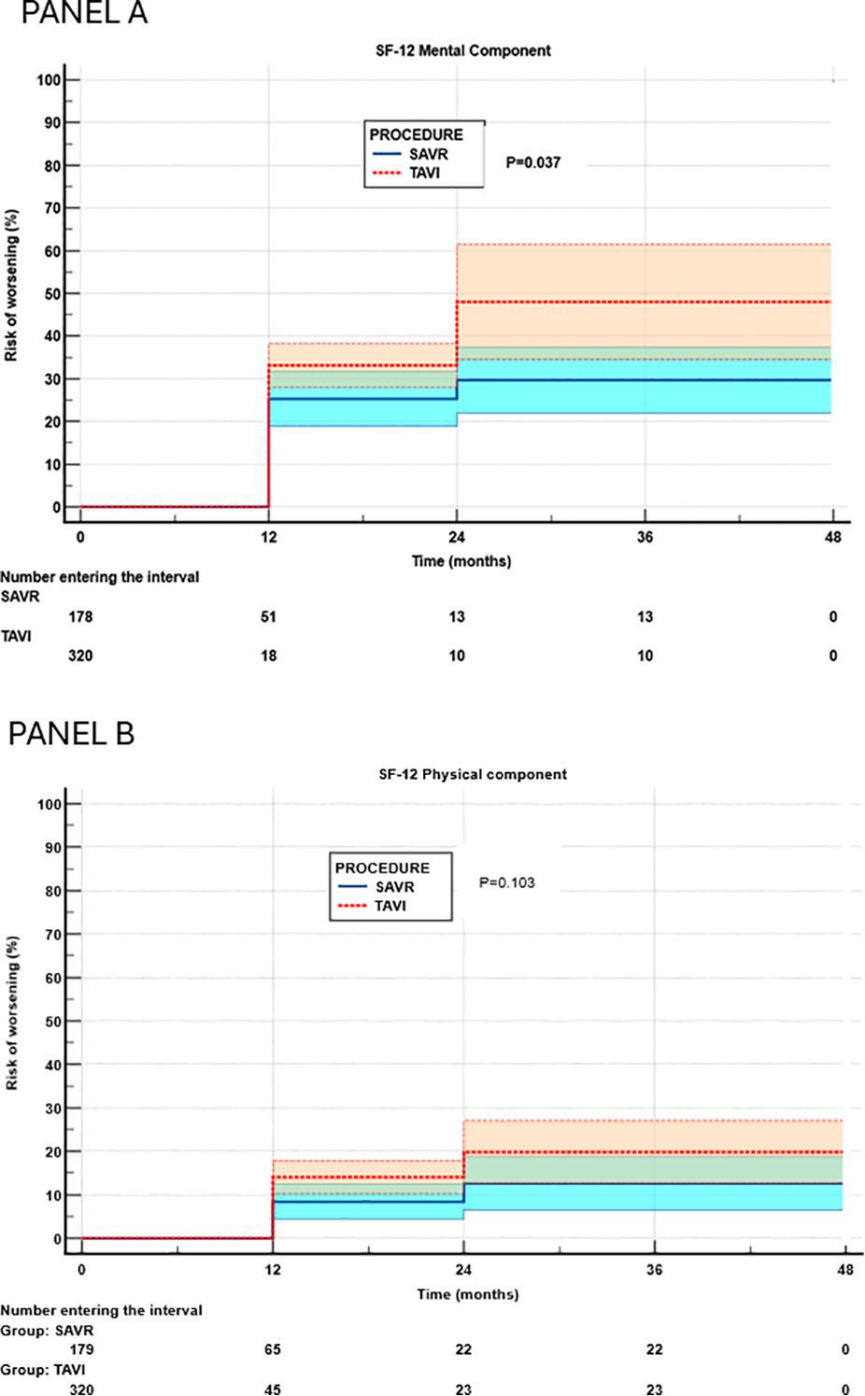
Risk of worsening of mental **(A)** and physical **(B)** components of health-related quality of life (Kaplan–Meier analysis). SAVR, surgical aortic valve replacement; TAVI, transcatheter aortic valve implantation.

Figure 2 reports the results of the cumulative risk of HRQL worsening assessed by Kaplan–Meier analysis for the mental (Figure 2A) and physical (Figure 2B) components of the SF-12. In univariate analysis, the TAVI group exhibited a significantly higher cumulative risk of deterioration in the mental component (hazards ratio 1.49, 95% confidence interval 1.03–2.20, *P* = 0.037), whereas no significant effect of TAVI was observed for the physical component (hazards ratio 1.49, 95% confidence interval 0.90–2.46, *P* = 0.123). After adjustment for potential confounders using Cox regression analysis (Supplementary Table S1), the association between risk of worsening of the mental domain and TAVI was no longer significant (hazards ratio 1.03, 95% confidence interval 0.68–1.56, *P* = 0.880), while TAVI was associated with a significantly (*P* = 0.045) higher risk of worsening in the physical component (hazards ratio 1.89, 95% confidence interval 1.02–3.56). No additional factors were significantly associated with worsening of either the SF-12 MCS or PCS.

Notably, the cumulative risk analysis of the SF-12 PCS showed a 6% absolute difference between groups at 12 months, while the analysis reported in Figure 1 showed a difference of only 1%. This discrepancy is due to the differences in the criteria used to adjudicate reliable worsening over time. Actually, in the SAVR group, seven of 31 patients (22.5%) recovered to a physical condition equal to or better than the baseline after an initial worsening, compared with seven of 60 patients (11.6%) in the TAVI group.

### Propensity-matched groups

**Figure 3:**
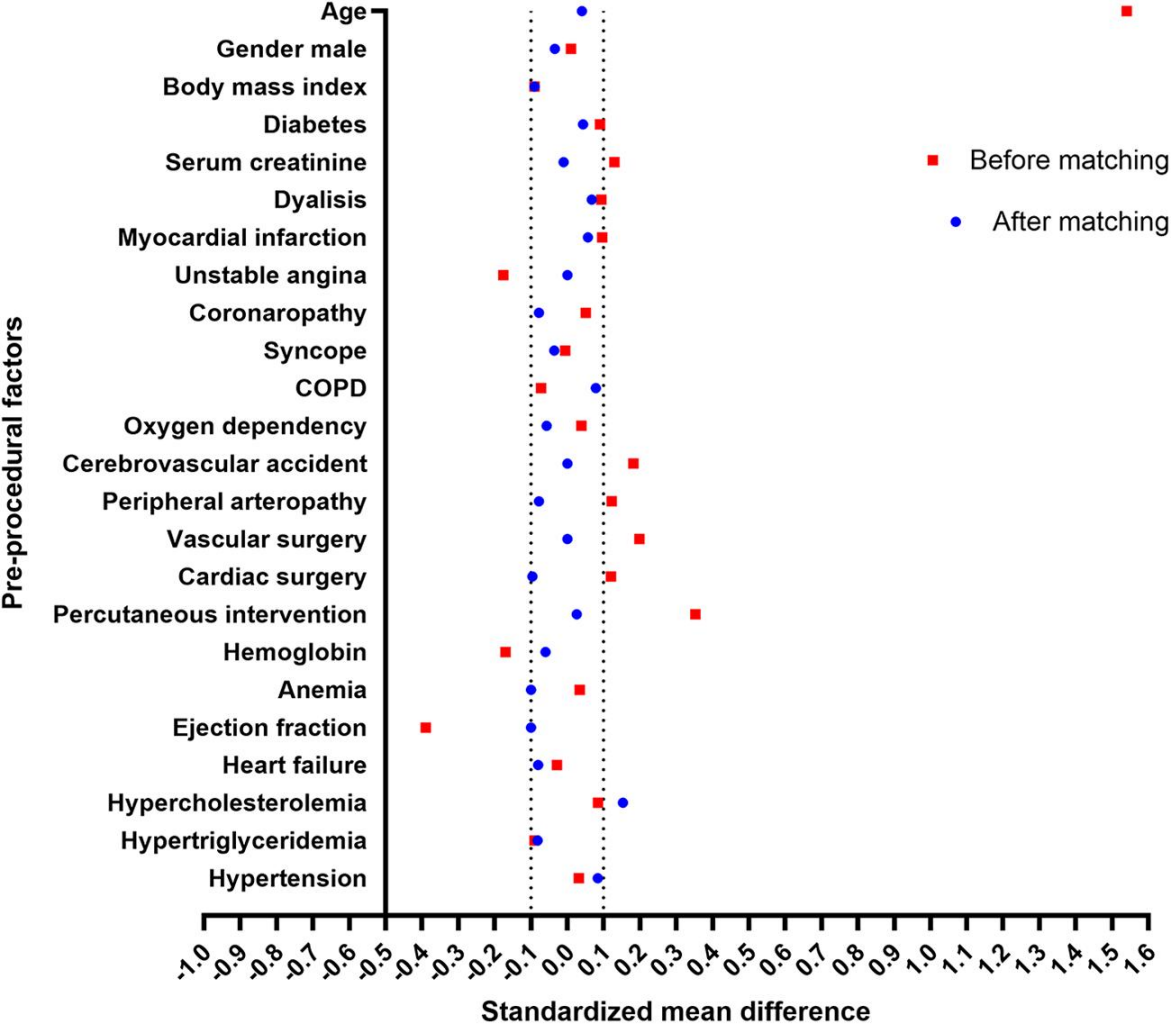
Standardized mean differences between surgical aortic valve replacement and transcatheter aortic valve implantation groups before and after propensity matching. COPD, chronic obstructive pulmonary disease.

Two propensity-matched groups were created, comprising 92 patients who underwent TAVI and 92 patients who underwent SAVR. Variables included in the propensity regression equation were those that showed a statistically significant difference between the SAVR and TAVI groups at *P* < 0.1. These variables included age, body mass index, ejection fraction, unstable angina, prior cerebrovascular accident, prior vascular surgery, prior percutaneous coronary intervention, and the use of angiotensin-converting enzyme (ACE) inhibitors, statins, P2Y12 antiplatelet agents, and direct oral anticoagulants. Group matching (Figure 3) was satisfactory, with SMDs not exceeding 0.1 for any of the 24 variables considered for checking the reliability of the propensity score matching, with the only exception of hypercholesterolemia (SMD 0.153). Notably, the most relevant variable being different in the overall population was age (73.3 ± 4.8 years in the SAVR group and 81.3 ± 5.5 years in the TAVI group, *P* = 0.001); this imbalance was almost perfectly corrected in the propensity-matched subgroup (75.9 ± 4.3 years in the SAVR group and 76.1 ± 4.9 years in the TAVI group, *P* = 0.810).

#### One-year follow-up

**Figure 4:**
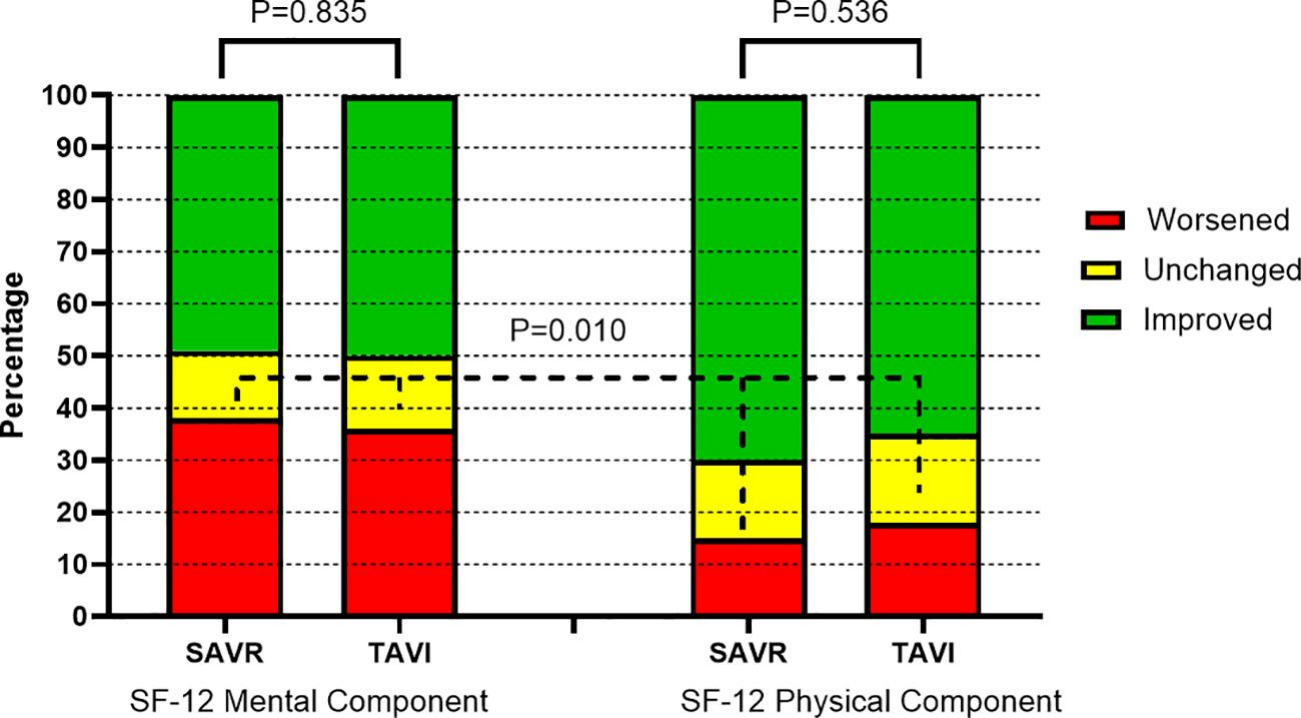
One-year changes in mental and physical components of health-related quality of life (postmatching). SAVR, surgical aortic valve replacement; TAVI, transcatheter aortic valve implantation.

Changes in HRQL, as defined by the RCI, in the SAVR and TAVI groups are shown in Figure 4. Overall, the changes were not significantly different between the two groups; a reliable worsening of the SF-12 MCS was observed in 35.6% of SAVR patients and 37.8% of TAVI patients, while the SF-12 PCS showed a significantly (*P* = 0.010) lower rate of worsening (17.8% in the SAVR group and 14.9% in the TAVI group) compared with the SF-12 MCS.

#### Long-term follow-up

**Figure 5:**
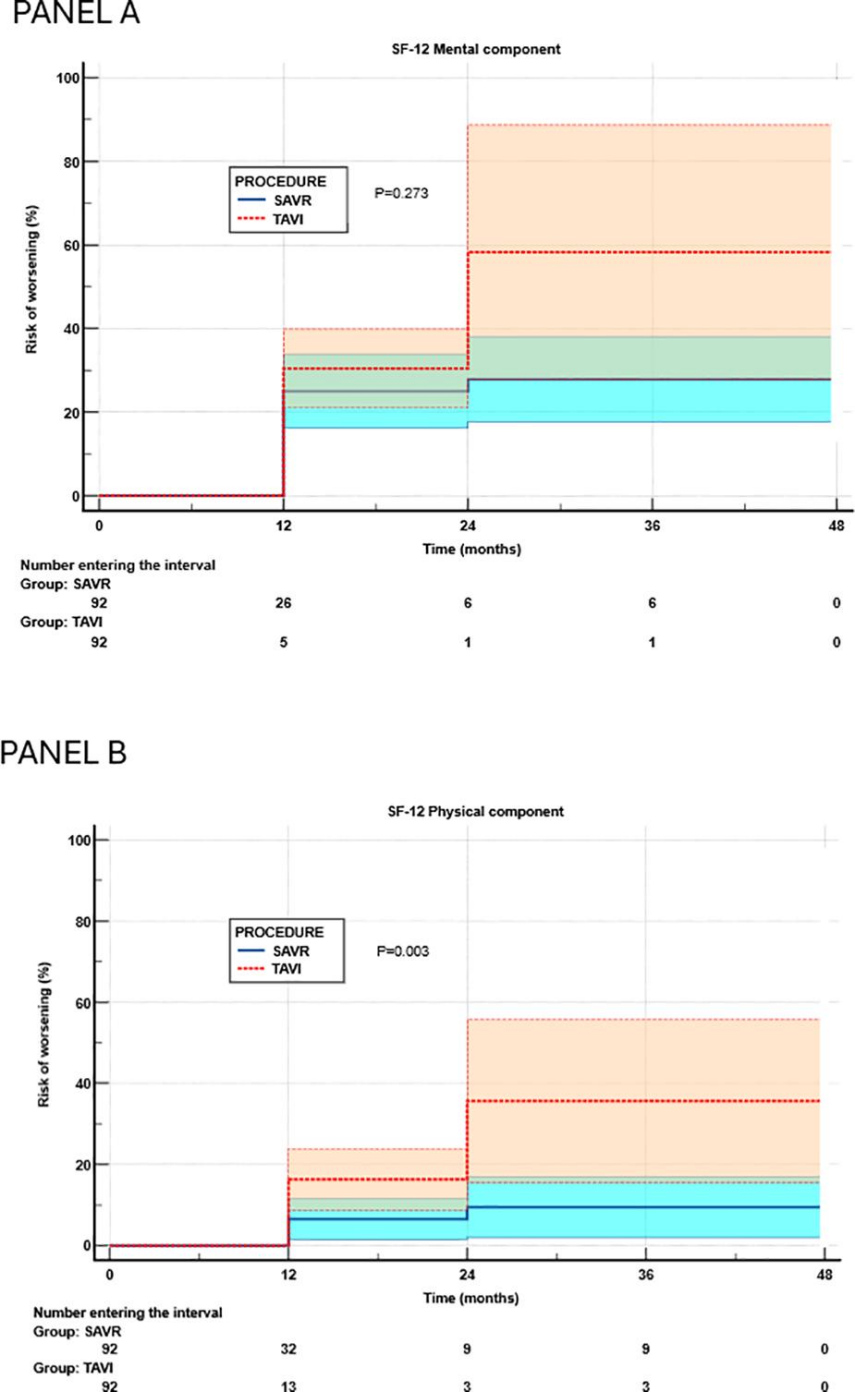
Risk of worsening of mental **(A)** and physical **(B)** components of health-related quality of life in propensity-matched groups (Kaplan–Meier analysis). SAVR, surgical aortic valve replacement; TAVI, transcatheter aortic valve implantation.

Figure 5 reports the Kaplan–Meier estimates of cumulative risk of HRQL worsening in the mental (Figure 5A) and physical (Figure 5B) domains. No significant between-group difference was observed in the SF-12 MCS (TAVI hazards ratio 1.42, 95% confidence interval 0.76–2.64, *P* = 0.273), whereas the risk of worsening of the SF-12 PCS was significantly higher in the TAVI group (hazards ratio 3.53, 95% confidence interval 1.54–8.06, *P* = 0.003).

A Cox regression analysis adjusting for potential confounders was performed within the propensity-matched groups (Supplementary Table S2). Several preoperative variables were included as potential confounders in this analysis. Worsening of the SF-12 MCS was not associated with TAVI (hazards ratio 1.23, 95% confidence interval 0.72–2.11, *P* = 0.446). Chronic obstructive pulmonary disease (COPD) emerged as the only independent predictor of SF-12 MCS worsening (hazards ratio 2.26, 95% confidence interval 1.15–4.45, *P* = 0.017). Conversely, after adjustment for confounders, TAVI remained independently associated with worsening of the SF-12 PCS (hazards ratio 3.91, 95% confidence interval 1.46–10.5, *P* = 0.007), and no other factors were independently associated with this event.

### Early cognitive function and 1-year HRQL

**Figure 6:**
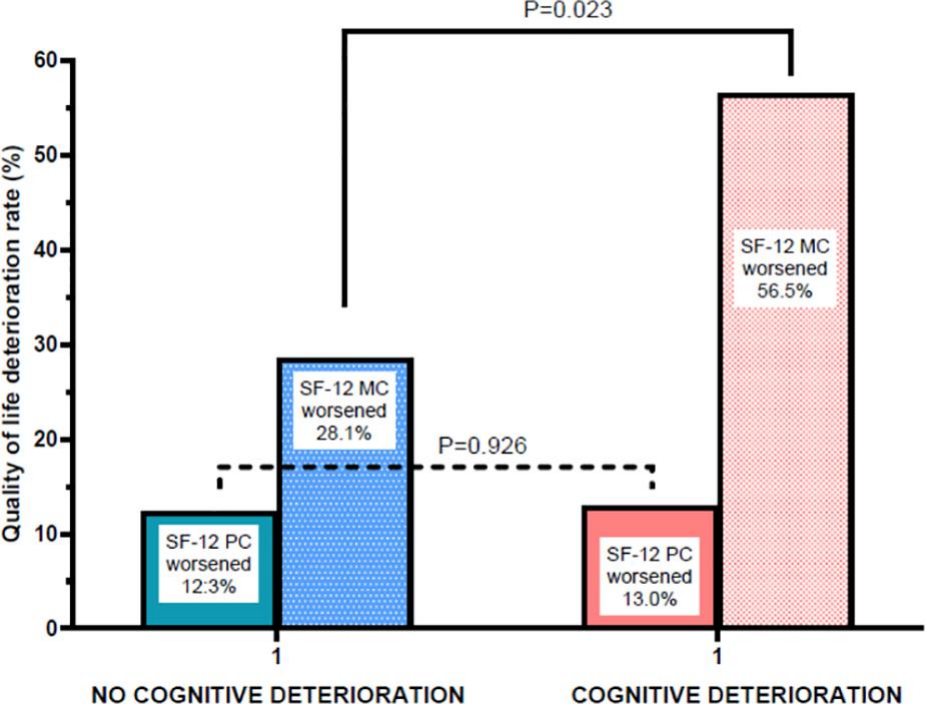
Changes in mental (MC) and physical (PC) components of health-related quality of life at 1-year follow-up in patients with or without early neurocognitive deterioration.

A subgroup of 80 patients (57 TAVI and 23 SAVR) underwent assessment of cognitive function at 2–3 months after the procedure and HRQL assessment at 12 months (Figure 6 and Supplementary Table S3). No association was observed between early cognitive deterioration and worsening of the SF-12 PCS at 12 months; conversely, a significant association (*P* = 0.023) was observed between early cognitive function deterioration and SF-12 MCS worsening at 12 months, with a relative risk for SF-12 MCS worsening of 3.2 (95% confidence interval 1.18–8.94) in patients who experienced cognitive decline 9–10 months earlier. This relationship was not affected by the type of procedure (TAVI or SAVR).

## Discussion

The main results of our study are as follows:

a. At 1 year after the procedure, no differences were observed in HRQL between SAVR and TAVI in both the mental and physical domains, with similar proportions of patients showing improvement, no change, or deterioration. In both groups, the incidence of worsening was higher for the mental component than for the physical component of HRQL.

In absolute terms, there was a significant improvement in the HRQL mental and physical components, which ultimately resulted in comparable values in the SAVR and TAVI groups. These results in the overall patient population were confirmed in the propensity-matched groups.

b. The risk of worsening of HRQL during follow-up, when adjusted for potential confounders, was similar for the mental component but lower for the physical component in the SAVR group. This finding was confirmed by a propensity matching of SAVR and TAVI.
c. Patients who experienced a clinically relevant worsening of cognitive function shortly (2–3 months) after the procedure were at higher risk of long-term (1 year) deterioration in the mental component of HRQL.

PROs provide valuable insights into patients' perceptions of their health status and of changes related to therapeutic maneuvers. A recent statement by the European Society of Cardiology highlights the role of PROs in cardiovascular disease, recognizing them as tools for assessing the efficacy of treatments and interventions (17).

Previous studies have addressed changes in HRQL after SAVR and TAVI procedures. Amonn and colleagues (7) examined a selected population of high-risk patients undergoing transapical TAVI compared with those receiving SAVR, with treatment allocation decided by the heart team. HRQL was assessed before treatment and at a mean follow-up of 15 months (with a high standard deviation of 10 months). One hundred patients completed follow-up, and no differences were observed between groups in terms of mental health and other scales of the SF-36; however, the general health subscale was significantly lower in the TAVI group. Given the large difference in baseline conditions, this difference was no longer significant after adjustment for potential confounders. Surman and colleagues (8) investigated three groups of patients undergoing SAVR, TAVI, or coronary surgery, for a total of 331patients consecutively enrolled at a single institution. At 12-month follow-up, no differences in HRQL were found among the groups. Tokarek and colleagues (9) conducted a prospective study including 173 patients divided into four groups: SAVR (via sternotomy, mini-sternotomy, or mini-thoracotomy) and TAVI. Allocation to TAVI or SAVR was based on clinical decision-making. At 12-month follow-up, HRQL was significantly better in the TAVI group compared with all SAVR subgroups. Baron and colleagues (3) analyzed 1,000 low-risk patients randomized to TAVI or SAVR using the Kansas City Cardiomyopathy Questionnaire-Overall Summary (KCCQ), the SF-36, and the EuroQoL instruments. Follow-up evaluations at 1, 6, and 12 months showed a higher rate of excellent outcomes (survival without a significant decline in quality of life) in the TAVI arm. Finally, the most important study (10) was a randomized controlled trial that included 2,032 patients and had a follow-up period of 5 years. In this trial, quality of life (assessed using the KCCQ) did not differ significantly between SAVR and TAVI at 5-year follow-up or at any intermediate time point.

Our results are in line with these previous studies with regard to the mental component of HRQL; conversely, we observed a higher rate of worsening of the physical component in the TAVI group. Various possible interpretations may account for this difference.

a. All previous studies have addressed changes in HRQL using a continuous variable (points). Conversely, we applied the RCI, a well-validated tool often used in psychological studies to determine the clinical relevance of changes. This dichotomization, based on an objective statistical criterion, allowed us to perform adequate analyses based on the cumulative risk of HRQL worsening and to account for the possibility that worsening might be transient. Actually, this is what happened to the SAVR group, which had a recovery rate almost twice that of the TAVI group. As a result, despite similar rates of events at 12 months, the cumulative risk was significantly lower in the SAVR group. It could be argued that, years after the procedure, TAVI patients might have experienced the effects of older age as a determinant of a greater decrease in HRQL in the physical domain. However, even in a propensity-matched analysis in which both groups had the same age at the time of procedure, the results obtained in the overall patient population were confirmed.
b. With the exception of one study, follow-up periods in previous reports were in the range of 12–15 months. Not surprisingly, at this relatively short follow-up, our results are consistent with those of previous studies. It is likely that the SAVR group suffered more the effects of surgery in the early months, subsequently recovering better than the TAVI group.
c. Our patient population is representative of a real-world scenario, where all kinds of patients are included, regardless of the severity of the baseline profile. Conversely, the other studies have focused on selected patient populations, with selection criteria generally based on the preprocedural profile (low, intermediate, and high risk). In particular, the previously cited large randomized trial (10) included only low-risk patients. In that population, the baseline KCCQ-OS score has a mean of around 54 points in both groups, corresponding to fair-to-good quality of life (18). In our series, the baseline SF-12 PCS has a mean value of 40.7 points, corresponding to a value below the 25th percentile of the general population (19), indicating a considerable degree of deterioration. It is therefore likely that a low-risk patient population might not be a sensitive environment for detecting postprocedural changes.

It must be admitted, however, that the large number of patients lost to follow-up, albeit in line with other studies, makes it difficult to state with absolute certainty that the physical component of SF-12 is significantly more prone to deterioration in TAVI patients.

An additional finding of our study is that patients who experience early cognitive deterioration are more likely to develop worsening of the mental component of HRQL at 1-year follow-up compared with those without cognitive deterioration. The present study was not designed to address the role of SAVR and TAVI in determining cognitive dysfunction. Briefly, SAVR has been associated with air micro-embolism and the deleterious effects of cardiopulmonary bypass, whereas TAVI is associated with embolization of calcium debris. These mechanisms have already been addressed in a previous study (14). A potentially important consequence of our finding is that patients with neurocognitive dysfunction may benefit from targeted neurocognitive rehabilitation aimed at preventing long-term, probably irreversible worsening of the mental component of HRQL. Therefore, a routine cognitive assessment may be considered for patients undergoing both SAVR and TAVI. Notably, the relationship between early cognitive dysfunction and worsening of the SF-12 MCS at 1 year remains unaffected by the type of procedure (SAVR or TAVI).

There are limitations in our study. An important one is the proportion of patients lost to follow-up, which was 38%. However, loss to follow-up proportions of 30% are quite common in non-randomized studies comparing SAVR and TAVI (7). Notably, the proportion of patients lost to follow-up was higher in the TAVI group, probably due to the older age in this group. A second limitation is the relatively low sample size of the subgroup receiving both MOCA and SF-12 after the first year of follow-up. The propensity matching method may suffer from the common problem of missing variables, despite 23 variables being considered. The strength of our study lies in the assessment of the clinical relevance of changes in HRQL and neurocognitive function using an objective and well-validated tool (RCI).

In conclusion, our study challenges the concept of interchangeability between SAVR and TAVI and highlights the importance of life expectancy in the decision-making process. Extending the observation period beyond 1 year appears essential to reveal the potential long-term effects of one procedure compared to the other. Therefore, the more restrictive European approach, particularly with respect to age thresholds for referral of patients to TAVI, presently appears more adequate than the more liberal strategy advocated by US guidelines.

## Data Availability

The raw data supporting the conclusions of this article will be made available by the authors, without undue reservation.
