# Supplementary material for: Trajectory of cognitive function and quality of life following stenotic aortic valve procedures
Source: Front Cardiovasc Med. 2026 Feb 6;13:1659733. doi: 10.3389/fcvm.2026.1659733 (PMC12920561; doi:10.3389/fcvm.2026.1659733)
Supplement: Supplementary file 1 [file Table1.docx]

**Supplementary table 1. Cox regression analysis in the pre-matching population**

1. Hazard ratio for SF-12 worsening of mental component

| Covariate | b | SE | Wald | P | HR | 95% CI of HR |
| --- | --- | --- | --- | --- | --- | --- |
| TAVI | 0,03162 | 0,2096 | 0,02275 | 0,8801 | 1,0321 | 0,6844 to 1,5566 |
| Age | 0,01198 | 0,01513 | 0,6270 | 0,4284 | 1,0121 | 0,9825 to 1,0425 |
| Body mass index | -0,009407 | 0,01815 | 0,2687 | 0,6042 | 0,9906 | 0,9560 to 1,0265 |
| Ejection fraction | -0,007174 | 0,008043 | 0,7955 | 0,3724 | 0,9929 | 0,9773 to 1,0086 |
| Unstable angina | -0,4077 | 0,7161 | 0,3241 | 0,5691 | 0,6652 | 0,1635 to 2,7071 |
| COPD | 0,3936 | 0,2337 | 2,8362 | 0,0922 | 1,4822 | 0,9376 to 2,3434 |
| Creatinine | 0,1008 | 0,05926 | 2,8918 | 0,0890 | 1,1060 | 0,9847 to 1,2423 |
| Diabetes | -0,02401 | 0,1909 | 0,01583 | 0,8999 | 0,9763 | 0,6716 to 1,4192 |
| Previous PCI | 0,1891 | 0,2078 | 0,8285 | 0,3627 | 1,2082 | 0,8040 to 1,8155 |
| Hemoglobin | -0,01873 | 0,04841 | 0,1498 | 0,6987 | 0,9814 | 0,8926 to 1,0791 |
| Stroke | 0,04660 | 0,3186 | 0,02139 | 0,8837 | 1,0477 | 0,5611 to 1,9564 |

1. Hazard ratio for SF-12 worsening of physical component

| Covariate | b | SE | Wald | P | HR | 95% CI of HR |
| --- | --- | --- | --- | --- | --- | --- |
| TAVI | 0,6375 | 0,3266 | 3,8102 | 0,0453 | 1,8917 | 1.0232 to 3,5880 |
| Age | -0,03194 | 0,02223 | 2,0645 | 0,1508 | 0,9686 | 0,9273 to 1,0117 |
| Body mass index | -0,05240 | 0,03121 | 2,8198 | 0,0931 | 0,9489 | 0,8926 to 1,0088 |
| Ejection fraction | 0,004275 | 0,01273 | 0,1128 | 0,7370 | 1,0043 | 0,9795 to 1,0297 |
| Unstable angina | -12,3778 | 281,6599 | 0,001931 | 0,9649 | 0,0000 | 7,4247E-246 to 2,3884E+234 |
| COPD | -0,8393 | 0,5960 | 1,9831 | 0,1591 | 0,4320 | 0,1343 to 1,3893 |
| Creatinine | 0,03048 | 0,1215 | 0,06292 | 0,8019 | 1,0309 | 0,8125 to 1,3081 |
| Diabetes | -0,1177 | 0,2940 | 0,1602 | 0,6889 | 0,8890 | 0,4996 to 1,5817 |
| Previous PCI | -0,1247 | 0,3536 | 0,1243 | 0,7244 | 0,8828 | 0,4415 to 1,7653 |
| Hemoglobin | -0,06822 | 0,07463 | 0,8357 | 0,3606 | 0,9341 | 0,8069 to 1,0812 |
| Stroke | -0,1731 | 0,5235 | 0,1094 | 0,7409 | 0,8411 | 0,3015 to 2,3464 |

CI: confidence interval; Baseline cumulative hazard function [[Show]](javascript:showdiv('d20','d21','table1');)COPD: chronic obstructive pulmonary disease; HR: hazard ratio; PCI: percutaneous coronary intervention

**Baseline cumulative hazard function** [**[Show]**](javascript:showdiv('d20','d21','table1');)

**Baseline cumulative hazard function** [[Show]](javascript:showdiv('d18','d19','table1');)
