# Supplementary material for: Trajectory of cognitive function and quality of life following stenotic aortic valve procedures
Source: Front Cardiovasc Med. 2026 Feb 6;13:1659733. doi: 10.3389/fcvm.2026.1659733 (PMC12920561; doi:10.3389/fcvm.2026.1659733)
Supplement: Supplementary file 2 [file Table2.docx]

**Supplementary table 2. Cox regression analysis in the post-matching population**

1. Hazard ratio for SF-12 worsening of mental component

| Covariate | b | SE | Wald | P | HR | 95% CI of HR |
| --- | --- | --- | --- | --- | --- | --- |
| TAVI | 0,1337 | 0,2859 | 0,2186 | 0,6401 | 1,1430 | 0,6527 to 2,0016 |
| Age | -0,0003006 | 0,03501 | 0,00007369 | 0,9932 | 0,9997 | 0,9334 to 1,0707 |
| Body mass index | -0,02761 | 0,02941 | 0,8813 | 0,3478 | 0,9728 | 0,9183 to 1,0305 |
| Ejection fraction | -0,01076 | 0,01527 | 0,4960 | 0,4812 | 0,9893 | 0,9601 to 1,0194 |
| Unstable angina | -12,5809 | 287,1804 | 0,001919 | 0,9651 | 0,0000 | 1,2116E-250 to 97,5117E+237 |
| COPD | 0,8176 | 0,3442 | 5,6409 | 0,0175 | 2,2651 | 1,1536 to 4,4474 |
| Creatinine | 0,04817 | 0,09582 | 0,2527 | 0,6152 | 1,0493 | 0,8697 to 1,2661 |
| Diabetes | -0,1618 | 0,3427 | 0,2230 | 0,6368 | 0,8506 | 0,4345 to 1,6650 |
| Previous PCI | 0,5032 | 0,3691 | 1,8587 | 0,1728 | 1,6540 | 0,8023 to 3,4099 |
| Hemoglobin | -0,04652 | 0,07972 | 0,3404 | 0,5596 | 0,9545 | 0,8165 to 1,1160 |
| Stroke | 0,6455 | 0,4867 | 1,7589 | 0,1848 | 1,9070 | 0,7346 to 4,9506 |
|  |  |  |  |  |  |  |

1. Hazard ratio for SF-12 worsening of physical component

| Covariate | b |  | SE | Wald | P | HR | 95% CI of HR |
| --- | --- | --- | --- | --- | --- | --- | --- |
| TAVI | 1,3636 |  | 0,5042 | 7,3143 | 0,0068 | 3,9102 | 1,4555 to 10,5045 |
| Age | -0,04985 |  | 0,05623 | 0,7861 | 0,3753 | 0,9514 | 0,8521 to 1,0622 |
| Body mass index | -0,07514 |  | 0,05246 | 2,0517 | 0,1520 | 0,9276 | 0,8370 to 1,0281 |
| Ejection fraction | 0,04179 |  | 0,02657 | 2,4740 | 0,1157 | 1,0427 | 0,9898 to 1,0984 |
| Unstable angina | -12,7342 |  | 513,2496 | 0,0006156 | 0,9802 | 0,0000 | 0,0000 to 10,1423E+303 |
| COPD | -12,5822 |  | 201,7229 | 0,003891 | 0,9503 | 0,0000 | 6,6925E-178 to 17,6042E+165 |
| Creatinine | -0,001245 |  | 0,2358 | 0,00002790 | 0,9958 | 0,9988 | 0,6291 to 1,5855 |
| Diabetes | -0,8307 |  | 0,6404 | 1,6823 | 0,1946 | 0,4357 | 0,1242 to 1,5290 |
| Previous PCI | -0,1512 |  | 0,7552 | 0,04007 | 0,8413 | 0,8597 | 0,1956 to 3,7776 |
| Hemoglobin | 0,06672 |  | 0,1423 | 0,2199 | 0,6391 | 1,0690 | 0,8088 to 1,4128 |
| Stroke | -0,6881 |  | 1,0426 | 0,4356 | 0,5093 | 0,5025 | 0,0651 to 3,8785 |

CI: confidence interval; Baseline cumulative hazard function [[Show]](javascript:showdiv('d20','d21','table1');)COPD: chronic obstructive pulmonary disease; HR: hazard ratio; PCI: percutaneous coronary intervention
