# Supplementary material for: Trajectory of cognitive function and quality of life following stenotic aortic valve procedures
Source: Front Cardiovasc Med. 2026 Feb 6;13:1659733. doi: 10.3389/fcvm.2026.1659733 (PMC12920561; doi:10.3389/fcvm.2026.1659733)
Supplement: Supplementary file 3 [file Table3.docx]

Supplementary table 3: Pre-procedural pattern of the population receiving both quality of life and neurocognitive tests.

______________________________________________________________________

Variable Overall SAVR TAVI P

(N=80) (N=23) (N=57)

_______________________________________________________________________

Demographics

Age (years) 79.5 (6.7) 73.3 (4.8) 82 (5.7) 0.001

Gender male 36 (45) 9 (39.1) 27 (47.4) 0.503

Body mass index (kg/m^2^) 27.1 (4.5) 27.5 (4.3) 26.9 (4.5) 0.583

Baseline conditions

Haemoglobin (g/dL) 13 (1.5) 13.5 (1.5) 12.9 (1.5) 0.101

Left ventricular ejection fraction 58.4 (10.6) 61.6 (9.4) 57.2 (11.6) 0.117

Serum creatinine (mg/dL) 1.08 (0.76) 0.9 (0.34) 1.15 (0.86) 0.182

Comorbidities

Arterial hypertension 59 (73.8) 17 (73.9) 42 (73.7) 0.983

Diabetes 30 (37.5) 4 (17.4) 26 (45.6) 0.018

Anemia 26 (32.5) 5 (21.7) 21 (36.8) 0.192

Dialysis 2 (2.5) 0 (0) 2 (3.5) 0.363

Previous myocardial infarction 13 (16.3) 2 (8.7) 11 (19.3) 0.245

Unstable angina 1 (1.3) 0 (0) 1 (1.8) 0.523

Coronaropathy 8 (10) 3 (13) 5 (8.8) 0.564

Congestive heart failure 9 (11.3) 1 (4.3) 8 (14) 0.215

Ipercholesterolemia 6 (7.5) 3 (13) 3 (5.3) 0.408

Ipertrigliceridemia 1 (1.3) 1 (4.3) 0 (0) 0.235

Syncope 11 (13.8) 4 (17.4) 7 (12.3) 0.548

COPD 12 (15) 4 (17.4) 8 (14) 0.704

Oxygen dependency 3 (3.8) 1 (4.3) 2 (3.6) 0.882

Cerebrovascular accident 10 (12.5) 2 (8.7) 8 (14) 0.513

Previous vascular surgery 2 (2.5) 0 (0) 2 (3.5) 0.363

Previous cardiac surgery 3 (3.8) 1 (4.3) 2 (3.5) 0.858

Previous PCI 20 (25) 4 (17.4) 16 (28.1) 0.318

Quality of life

SF-12 mental component 48.8 (9.6) 49.7 (9.1) 47.8 (10.1) 0.171

SF-12 physical component 40.8 (9.8) 41.4 (10.4) 40.3 (9.2) 0.423

_______________________________________________________________________

Data is number (%) or mean (standard deviation); COPD: chronic obstructive pulmonary disease; PCI: percutaneous intervention; SAVR: surgical aortic valve replacement; TAVI: transcatheter aortic valve implantation.
